# Interdisciplinary Strategies for Improving Oral Health in Older Adults: A Comprehensive Review

**DOI:** 10.3390/geriatrics11010022

**Published:** 2026-02-19

**Authors:** Joanna Cheuk Yan Hui, Lindsey Lingxi Hu, Alice Kit Ying Chan, Chun Hung Chu

**Affiliations:** Faculty of Dentistry, The University of Hong Kong, Hong Kong 999077, China; cyjaonna@hku.hk (J.C.Y.H.); hu_lingxi@connect.hku.hk (L.L.H.); dralice@hku.hk (A.K.Y.C.)

**Keywords:** older adults, elderly, oral health, public health, prevention, health strategies

## Abstract

Oral health in older adults is a critical component of overall well-being requiring integrated, interdisciplinary approaches to address its complex interplay of medical, functional, and psychosocial challenges. The aim of this is to examine strategies to enhance interdisciplinary collaboration among dental professionals, physicians, nurses, nutritionists, and caregivers to improve oral health outcomes in aging populations. Older adults commonly face dental problems such as periodontal disease which can be exacerbated by polypharmacy, systemic diseases, and barriers to accessing care. These multifaceted needs necessitate coordinated efforts across dentistry, geriatric medicine, nursing, and social support systems. Strategies of effective interdisciplinary care include: (1) Medical-dental integration, enabling physicians to screen for oral health issues during routine assessments; (2) Nursing and caregiver engagement in daily oral hygiene support and early problem identification; (3) Nutritional interventions tailored to address chewing difficulties and prevent malnutrition; (4) Social support systems to improve access to affordable care; and (5) Technology-driven solutions such as tele-dentistry to enhance communication, early detection, and care coordination. Despite these opportunities, systemic barriers persist, including fragmented healthcare systems, financial constraints, workforce shortages, cultural biases, and technological gaps. Progress requires commitment from policymakers, healthcare institutions, and health care professionals to prioritize geriatric oral health as a public health imperative. In conclusion, interdisciplinary collaboration enhances older adults’ oral-systemic health via cross-sector policies and healthcare workforce education. Implementing these strategies can mitigate oral health disparities, reduce the burden of chronic diseases, and improve quality of life for aging populations through holistic, patient-centered care.

## 1. Introduction

Oral health in older adults is a critical public health issue that intersects with broader medical, social, and economic concerns [1]. As global demographics shift toward an aging population—with the World Health Organization projecting that by 2050, one in six people worldwide will be over age 65—the need for effective geriatric oral healthcare models has become increasingly urgent [2,3]. This demographic transition brings complex oral health challenges that traditional dental care approaches are ill equipped to address, necessitating a paradigm shift toward interdisciplinary collaboration [4].

The oral-systemic health connection in older adults strongly supports the need for integrated care. Research has established bidirectional relationships between oral conditions and chronic diseases prevalent in aging populations [5]. For example, periodontal disease has been linked to poorer glycemic control in patients with diabetes, while severe tooth loss is associated with an increased risk of cardiovascular events [6]. These associations are further complicated by polypharmacy. The United States national data shows nearly 40% of adults aged ≥65 years use five or more prescription medications [7,8]. Many commonly used drugs can contribute to xerostomia (dry mouth), which is associated with increased caries risk due to loss of saliva’s protective effects [9,10]. The consequences of poor oral health extend beyond physical well-being; tooth loss and oral pain significantly impact nutritional intake, speech, self-esteem, and social interaction, contributing to depression and social isolation among vulnerable elders [11]. Current healthcare systems often fail older adults due to fragmented care delivery [12]. Dentistry remains siloed from general healthcare, creating significant gaps in treatment [5]. Primary care physicians frequently lack training to recognize oral health indicators, while dentists may be unaware of a patient’s complete medical history or medication regimen [13]. This disconnect is particularly problematic in institutional settings, where nursing staff—despite providing daily personal care—typically receive minimal oral health training [14]. The result is a perfect storm of unmet needs: untreated caries in 50% of community-dwelling seniors, periodontal disease in 70% of those over 65, and high rates of oral cancer mortality due to late detection [15].

Financial and structural barriers further compound these clinical challenges. In the United States, Medicare exclusion of routine dental care leaves nearly half of seniors without dental coverage, while Medicaid benefits vary widely by state [16]. Transportation limitations, physical disabilities, and cognitive impairments create additional obstacles to accessing care. Even when services are available, many older adults and their caregivers view dental problems as an inevitable consequence of aging rather than as treatable conditions, resulting in delayed disease detection and advanced disease [17]. The limitations of conventional dental models in addressing these multifaceted challenges are increasingly evident. Traditional practice structures focused on restorative procedures in clinical settings are often inadequate for homebound elders or those with dementia [18]. Fee-for-service reimbursement models discourage the preventive care and chronic disease management approaches essential for geriatric populations [19,20]. These systemic shortcomings demand innovative solutions that bridge professional disciplines and care settings. This review explores collaborative strategies among dental professionals, physicians, nurses, nutritionists, and caregivers to enhance oral health outcomes for older adults.

## 2. Methods

This review searched relevant English-language publications (original studies, clinical trials where available, observational studies, implementation reports, and review articles) indexed in MEDLINE and Google Scholar, supplemented by manual screening of reference lists from key articles. We reviewed reports from authoritative institutions, including the World Health Organization (WHO) and FDI World Dental Federation. We also analyzed U.S. policy documents from sources like the Centers for Medicare & Medicaid Services (CMS) for background and policy context. The last search date was 31 Dec 2025. Search terms were combined to capture three main concepts: older adults, oral health conditions, and interdisciplinary/integrated care models. Keywords included: “older adults”, “elderly”, “geriatric”; “oral health”, “dental care”, “oral diseases”, “periodontal disease”, “dental caries”, “xerostomia”, “tooth loss”, “oral cancer”; and “interdisciplinary”, “interprofessional”, “integrated care”, “medical–dental integration”, “primary care”, “nursing”, “caregiver”, “long-term care”, “nursing home”, “teledentistry”, “electronic health records”, and “Medicare dental”.

## 3. The Need for Interdisciplinary Care in Geriatric Oral Health

The oral health challenges faced by older adults are not isolated dental concerns but are deeply intertwined with systemic health, functional limitations, and socioeconomic factors [21]. This complex interplay necessitates an interdisciplinary approach that bridges dentistry with medicine, nursing, nutrition, and social care. Without coordinated efforts, older adults remain vulnerable to preventable oral diseases, systemic complications, and diminished quality of life [22].

### 3.1. The Oral-Systemic Health Connection in Aging

A growing body of evidence highlights the bidirectional relationship between oral health and chronic diseases in older adults [23]. Periodontal disease is not merely a localized gum infection but a chronic inflammatory condition that exacerbates systemic illnesses. Severe periodontitis is independently associated with a higher risk of cardiovascular diseases, including atherosclerosis and stroke, due to the systemic spread of oral pathogens and inflammatory mediators [24]. Similarly, diabetes and periodontal disease are intertwined: poor glycemic control worsens gum disease, while untreated periodontitis impairs blood sugar management [25]. Medication-induced xerostomia (dry mouth) further complicates oral health in older adults. Many commonly prescribed medications, including antihypertensives, antidepressants, and anticholinergics, reduce salivary flow, increasing susceptibility to dental caries, oral infections, and swallowing difficulties [26]. This is especially concerning given that nearly 90% of older adults take at prescription medication, with many on multiple drugs that compound oral health risks [27].

### 3.2. Functional and Cognitive Barriers to Oral Self-Care

Beyond medical comorbidities, physical and cognitive impairments significantly hinder oral hygiene maintenance [28]. Conditions such as arthritis, Parkinson’s disease, and stroke-related motor limitations make brushing and flossing difficult, leading to plaque accumulation and periodontal disease [27]. Studies show that older adults with severe dexterity issues are three times more likely to have untreated dental decay than those without such limitations [29]. Cognitive decline, particularly in Alzheimer’s disease and other dementias, presents additional challenges. Patients with advanced dementia may forget to brush their teeth, resist oral care due to confusion, or exhibit behaviours that complicate dental treatment [30]. Caregivers—whether family members or nursing home staff—often lack the training to manage these challenges, resulting in neglected oral hygiene and preventable conditions such as stomatitis or aspiration pneumonia [31].

### 3.3. Systemic Consequences of Poor Oral Health in Older Adults

The repercussions of untreated oral diseases extend far beyond the mouth. Aspiration pneumonia, a leading cause of death among frail elderly individuals, is strongly linked to poor oral hygiene; pathogenic bacteria from the oral cavity can be inhaled into the lungs, especially in bedridden patients or those with dysphagia [31]. Structured oral care programs in nursing homes can reduce aspiration pneumonia [32,33]. Malnutrition is another critical concern, as tooth loss, poorly fitting dentures, and oral pain often lead to inadequate nutrient intake [34]. Older adults with fewer than 20 teeth are at higher risk of protein-energy malnutrition, vitamin deficiencies, and frailty [35]. This creates a downward spiral: poor nutrition weakens immune function, making oral infections more severe, which in turn further impairs eating ability.

### 3.4. The Limitations of Siloed Healthcare Systems

Traditional healthcare models, in which dental and medical professionals operate independently, fail to address these interconnected issues [5]. Physicians managing chronic diseases such as diabetes or heart disease rarely assess oral health, while dentists may not have access to a patient’s full medical history or medication list. This fragmentation is especially problematic in long-term care facilities, where oral health is often deprioritized despite its impact on overall health [36]. Moreover, Medicare’s exclusion of routine dental care in the U.S. leaves many older adults without access to preventive services, forcing them to seek emergency care only when problems become severe [37]. Similar gaps exist in other healthcare systems where geriatric dentistry is not integrated into primary care.

### 3.5. The Imperative for Interdisciplinary Collaboration

**Figure 1:**
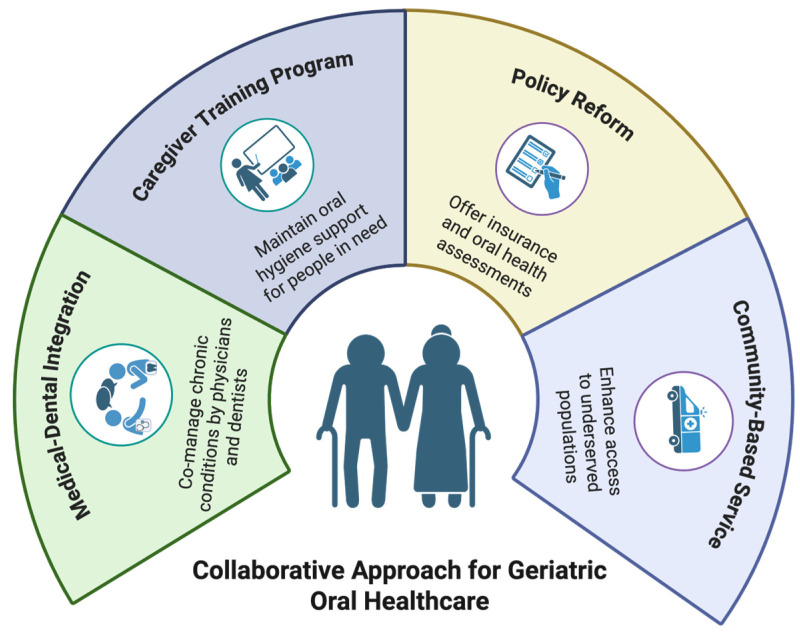
Collaborative Approach for Geriatric Oral Healthcare (Created with BioRender (2025) https://www.biorender.com, accessed on 1 January 2026).

Given these challenges, a collaborative approach is not just beneficial, it is essential. Effective geriatric oral healthcare requires medical-dental integration, caregiver training programs, policy reforms, and community-based interventions [5,38] (see Figure 1). By breaking down professional silos and fostering teamwork among healthcare providers, oral health disparities that disproportionately affect older adults can be mitigated. The following sections explore the key components of interdisciplinary models, barriers to implementation, and successful strategies for improving geriatric oral health outcomes.

## 4. Key Components of Interdisciplinary Oral Health Care

An effective interdisciplinary approach to geriatric oral healthcare requires coordinated efforts across multiple healthcare disciplines, each contributing unique expertise to address the complex needs of older adults [39]. This section details the essential components of such collaborative care models, highlighting roles, strategies, and evidence-based practices that can improve oral health outcomes for aging populations.

### 4.1. Medical-Dental Integration

**Figure 2:**
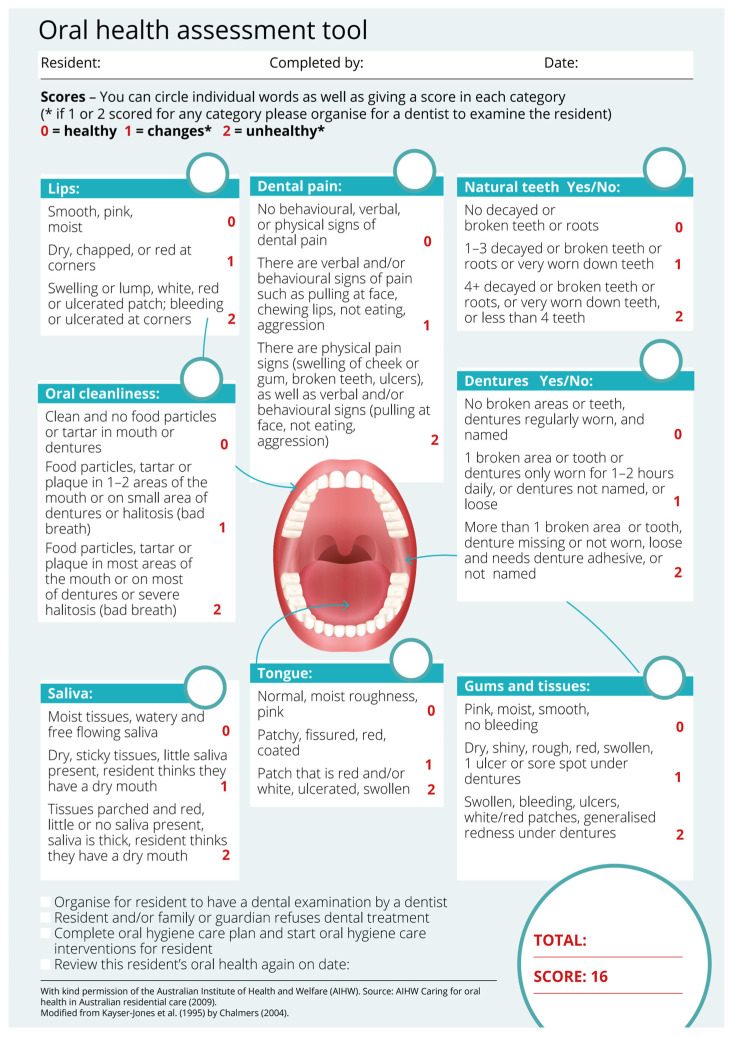
AIHW Oral Health Assessment Tool (Source: Australian Institute of Health and Welfare (AIHW) 2009 [41,42,47]).

The foundation of interdisciplinary oral healthcare lies in bridging the gap between medical and dental professionals [40]. Physicians—particularly geriatricians, internists, and primary care providers—play a crucial role in identifying oral health issues during routine check-ups. Figure 2 is the Oral Health Assessment Tool (OHAT) developed by the Australian Institute of Health and Welfare [41,42], OHAT can be incorporated into general health assessments to detect early signs of dental caries, periodontal disease, or oral lesions. Dentists must be informed about a patient’s medical history, including chronic conditions (e.g., diabetes, cardiovascular disease) and medications that impact oral health [43]. For example, bisphosphonates used for osteoporosis increase the risk of osteonecrosis of the jaw, necessitating preventive dental care before initiating treatment [44]. Similarly, patients on anticoagulants require careful management during dental procedures to avoid bleeding complications [45]. In integrated care models, physicians and dentists collaborate to adjust medications that cause xerostomia (e.g., substituting anticholinergics with alternatives that have fewer oral side effects), while dentists provide fluoride treatments or saliva substitutes to mitigate dry mouth symptoms [10,46].

### 4.2. Nursing and Caregiver Involvement in Daily Oral Hygiene

**Figure 3:**
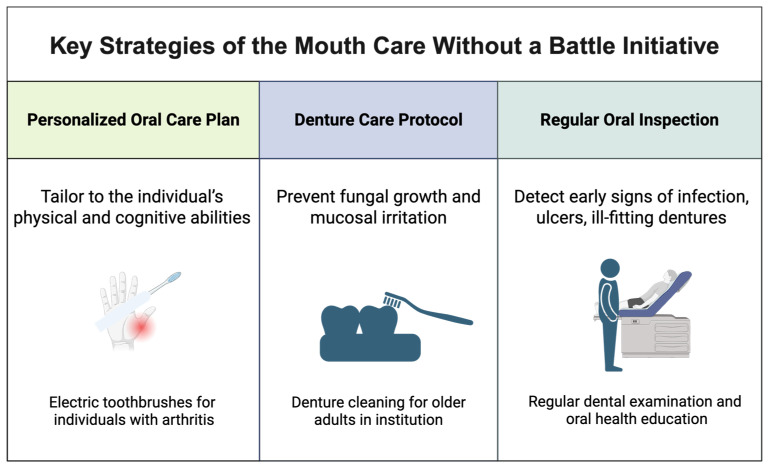
Key Strategies of the Mouth Care Without a Battle Initiative (Created with BioRender (2025) https://www.biorender.com, accessed on 1 January 2026).

Nurses and caregivers are often the frontline providers assisting older adults with daily oral hygiene, particularly in hospitals, nursing homes, and home care settings [48]. However, studies show that nursing staff frequently receive minimal training in oral care, leading to inconsistent practices. Structured training programs, such as the “Mouth Care Without a Battle” initiative, have been implemented to improve oral hygiene for people with dementia by teaching caregivers’ techniques to reduce resistance and ensure thorough cleaning [49]. Personalized oral care plans and denture care protocols are essential, and nurses should perform regular oral inspections to identify problems early. Figure 3 shows the key strategies of the mouth care without a battle initiative.

### 4.3. Nutritional Support for Oral and Systemic Health

Malnutrition and oral health are closely interconnected in older adults. Nutritionists and dietitians must work alongside dental teams to develop soft, nutrient-dense meal plans for patients with chewing difficulties or missing teeth. For example, protein-fortified purees, vitamin-rich soups, and hydration strategies can prevent weight loss and nutritional deficiencies while minimizing oral discomfort [34,50]. Dietary counselling should also address sugar intake, as older adults with dry mouth are at high risk for caries from sugary medications or snacks. Collaboration with pharmacists can help identify sugar-free alternatives for liquid medications.

### 4.4. Social Work and Community Health Worker Engagement

Many older adults face financial, transportation, or cultural barriers to dental care [21]. Social workers and community health workers can connect low-income seniors with sliding-scale clinics or charitable dental programs and assist with Medicaid applications or advocacy for expanded dental benefits under Medicare [17]. They also provide culturally sensitive education on oral hygiene in community centers and senior living facilities. Mobile dental units and teledentistry programs have proven effective in rural or underserved areas, where dentists can remotely assess oral conditions with the assistance of community health workers [51].

### 4.5. Technology and Innovation in Interdisciplinary Care

**Figure 4:**
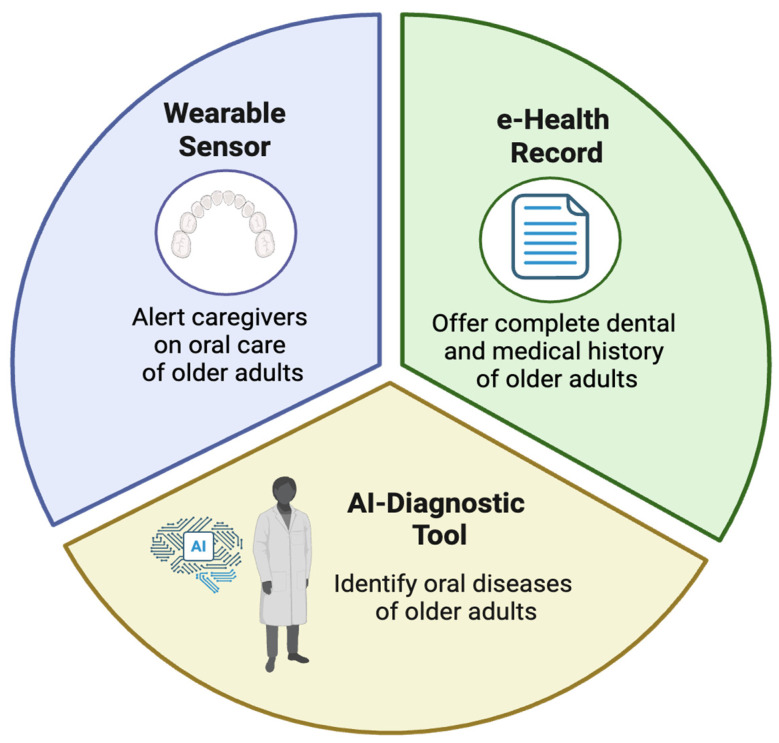
Technology and Innovation in Interdisciplinary Care (Created with BioRender (2025) https://www.biorender.com, accessed on 1 January 2026).

Technological advancements are revolutionizing interdisciplinary geriatric oral healthcare by breaking down traditional barriers between medical and dental providers [52] (see Figure 4). Integrated electronic health records (EHRs) enable seamless sharing of comprehensive patient data between physicians and dentists, ensuring all providers have access to complete medical histories, medication lists, and treatment plans [53]. Artificial intelligence is playing an increasingly vital role. Additionally, researchers are exploring AI-powered diagnostic tools and connected oral-care devices to support screening, monitoring, and communication in geriatric oral healthcare, potentially during routine examinations [52,54]. For patients with cognitive impairments, innovative wearable sensors and smart oral care devices can track brushing habits and oral hygiene routines, automatically alerting caregivers when assistance is needed. These technologies not only enhance early detection and intervention but also facilitate real-time communication between care team members, creating a truly integrated approach to managing the oral-systemic health connection in older adults [54]. By leveraging these digital solutions, healthcare systems can overcome traditional silos, improve care coordination, and deliver more proactive, personalized oral healthcare for aging populations.

### 4.6. Policy and Systemic Support

Successful implementation of interdisciplinary geriatric oral healthcare requires fundamental systemic reforms to overcome existing structural barriers [55]. Policy changes should include reimbursement reforms that incentivize medical-dental collaboration, such as establishing dedicated billing codes for oral health screenings conducted during primary care visits and expanding Medicare coverage to include essential dental services. Educational institutions and licensing bodies should mandate standardized geriatric oral health training across all healthcare professions, ensuring physicians, nurses, and allied health professionals possess core competencies in age-related oral health issues [56].

Regulatory frameworks and facility-level governance can support consistent oral hygiene protocols and staff training in institutional settings. These systemic changes must be supported by interoperable health information systems that facilitate seamless data sharing between medical and dental providers. Improving oral hygiene and oral health in institutionalized older adults is also associated with better health-related quality of life [57]. Addressing these policy gaps will create the necessary infrastructure to sustain interdisciplinary models that prioritize oral health as an integral component of comprehensive geriatric care.

Interdisciplinary oral healthcare for older adults is not a single intervention but a coordinated system in which dentists, physicians, nurses, nutritionists, and social workers each play a vital role [58]. By integrating medical and dental care, empowering caregivers, leveraging technology, and advocating for policy changes, we can address the multifaceted challenges of geriatric oral health. The next section examines the barriers to implementing these models and strategies to overcome them. Figure 4 shows the technology and Innovation in Interdisciplinary Care.

## 5. Challenges in Implementing Interdisciplinary Care

Despite the clear advantages of interdisciplinary approaches to geriatric oral health, translating these models into everyday practice presents considerable obstacles. Barriers arise at multiple levels—including systemic, financial, workforce, cultural, technological, and regulatory domains—impeding the widespread adoption of integrated care [5,59]. Understanding these challenges is essential for developing effective strategies to bridge gaps in service delivery and improve oral health outcomes for older adults.

### 5.1. Siloed Healthcare Systems

Despite the clear benefits of interdisciplinary approaches to geriatric oral healthcare, numerous systemic, financial, and cultural barriers hinder their widespread adoption. One of the most significant obstacles is the persistent fragmentation between medical and dental care systems, which operate under separate infrastructures, reimbursement models, and professional cultures. Dentists and physicians are often trained in isolation, resulting in gaps in communication and a lack of shared understanding about the oral-systemic health connection [60]. Many primary care providers receive minimal education on oral health during medical training, leading to missed opportunities for early intervention, while dentists may lack access to a patient’s complete medical history, including critical details about medications or chronic conditions [61]. This siloed approach is particularly problematic in long-term care facilities, where nursing staff—tasked with daily oral hygiene for residents—frequently lack proper training and protocols, leading to inconsistent and often inadequate care [59].

### 5.2. Financial Barriers

Financial barriers further complicate the implementation of interdisciplinary models. In the United States, Medicare does not cover routine dental care, leaving many older adults without access to preventive services and forcing them to delay treatment until emergencies arise [59,62]. Even when dental benefits are available through Medicaid or private insurance, reimbursement rates for geriatric oral healthcare are often insufficient to incentivize providers to treat this high-need population [63]. The economic burden disproportionately affects low-income seniors, racial and ethnic minorities, and rural residents, exacerbating existing health disparities [64,65]. Additionally, current billing structures seldom support collaborative care—physicians cannot bill for oral health screenings, and dentists are not compensated for coordinating with medical teams, creating a financial disincentive for interdisciplinary practice [66].

### 5.3. Workforce Shortages

Workforce shortages and uneven distribution of geriatric dental specialists present another major challenge. Many regions, particularly rural and underserved urban areas, lack dentists with specialized training in managing the complex needs of older adults, including those with cognitive impairments or multiple chronic conditions [67]. Even when providers are available, logistical barriers such as transportation difficulties, mobility limitations, and the high cost of dental visits prevent many seniors from accessing care [64,68,69]. Homebound older adults and nursing home residents face additional hurdles, as few dental professionals offer mobile or on-site services, leaving this vulnerable population reliant on overburdened caregivers for oral hygiene support [69,70].

### 5.4. Cultural and Attitudinal Barriers

Cultural and attitudinal barriers also impede progress. Oral health is frequently deprioritized in geriatric care, viewed as a cosmetic concern rather than a critical component of overall well-being [71]. Many older adults, particularly those from generations that did not grow up with preventive dental care, may not recognize the importance of regular dental visits or may accept tooth loss and oral pain as inevitable consequences of aging [72]. Caregivers, including family members and nursing home staff, often lack awareness of proper oral hygiene techniques or underestimate its impact on systemic health, leading to neglect until severe problems develop [73]. Furthermore, resistance to change within healthcare institutions can slow the adoption of new interdisciplinary models, as shifting long-established workflows and fostering collaboration across professions requires significant time, training, and institutional support [74].

### 5.5. Technological and Infrastructural Limitations

Technological and infrastructural limitations also pose challenges. While electronic health records (EHRs) have the potential to facilitate communication between medical and dental providers, many systems remain incompatible, preventing seamless data sharing [75]. Privacy regulations and differing documentation standards between medical and dental practices further complicate information exchange [75]. While teledentistry has emerged as a promising solution for reaching underserved populations, disparities in digital literacy and limited broadband access among older adults can restrict its effectiveness, particularly in rural or low-income communities [76].

### 5.6. Policy and Regulatory Hurdles

Policy and regulatory hurdles further constrain the expansion of interdisciplinary care. The lack of standardized oral health assessments in primary care settings means that many systemic conditions linked to poor oral health—such as diabetes complications or cardiovascular risks—go unaddressed [69,77]. Licensing restrictions may prevent dental hygienists from providing care in nursing homes or community settings without direct dentist supervision, limiting the scalability of preventive programs [70]. Additionally, the absence of national mandates for oral care in long-term care facilities results in inconsistent practices, with some institutions implementing rigorous protocols while others provide minimal attention to residents’ oral health needs [69,78].

Despite these challenges, opportunities for progress exist; pilot programs have demonstrated that interdisciplinary models can be implemented effectively with the right support. For example, hospital-based models such as perioperative oral management demonstrate how medical–dental collaboration can be implemented, with observational evidence suggesting associations with lower postoperative pneumonia rates [79]. Advocacy efforts to expand Medicare dental benefits are gaining momentum, and some regions have begun integrating oral health into Medicaid-managed care plans [73]. Interprofessional education initiatives are also helping bridge the gap between medical and dental training, fostering a new generation of providers who understand the value of collaboration [80,81].

Addressing these barriers will require coordinated efforts from policymakers, healthcare leaders, educators, and advocates. Reforming reimbursement structures to support team-based care, expanding geriatric dental training programs, and implementing standardized oral health protocols in all care settings are essential steps forward. By confronting these challenges directly, the healthcare system can move toward a future in which interdisciplinary oral healthcare is the norm rather than the exception, ensuring that older adults receive the comprehensive, patient-centered care they deserve.

## 6. Successful Models and Future Directions

Several healthcare systems have implemented interdisciplinary approaches that integrate oral assessment and professional oral care into broader care pathways for older adults. In Japan, perioperative oral management (POM) is a hospital-based model in which dental professionals collaborate with medical teams to screen for and manage oral conditions prior to surgery. Large-scale observational evidence suggests that structured POM approaches can be associated with lower postoperative pneumonia rates [79].

In the United States, delivery systems that operationalize medical–dental integration within a single organization can facilitate consistent coordination through shared infrastructure (e.g., aligned workflows, referral pathways, and preventive care protocols). For example, Kaiser Permanente Northwest (KPNW) offers an integrated health care system that includes both dental and medical care. Their medical–dental integration program in dental clinics was associated with higher odds of closing medical care gaps following dental visits, such as immunizations and chronic disease monitoring, demonstrating measurable benefits of integration beyond oral outcomes alone [82].

In long-term care settings, intensified oral care interventions combining daily assisted oral hygiene with professional oral care have been evaluated in nursing homes. Some trials have reported reduced pneumonia incidence among residents receiving enhanced oral care compared with usual care [32]. Caregiver training models (including structured approaches such as Mouth Care Without a Battle) may support implementation by improving staff skills, standardizing routines, and addressing dementia-related care resistance [49]. Although those training frameworks should be cited primarily for process improvement unless specific outcome evaluations are referenced, improving oral care delivery may also support resident comfort and oral hygiene–related outcomes.

Community-based interventions have also made strides in improving access to oral healthcare for underserved older adults. Mobile dental clinics, such as those operated by nonprofit organizations like the Pacific Dental Services (PDS) Foundation, bring preventive and restorative care directly to senior centers, low-income housing complexes, and rural areas, eliminating transportation barriers [83]. Partnerships between dental schools and community health centers have expanded capacity for geriatric dental services while providing hands-on training for future providers. Additionally, teledentistry platforms are bridging gaps in care by enabling remote consultations, particularly for homebound seniors or those in regions with dental provider shortages [73,84].

Future reforms must prioritize dental care financing structures that align with evidence-based integrated care models. The U.S. Medicare system exemplifies both the challenges and opportunities: while traditional Medicare excludes routine dental care (forcing beneficiaries into Medicare Advantage plans for coverage [85]), recent analyses reveal systemic limitations in these alternatives. Medicare Advantage plans exhibit wide variability in benefit adequacy [86] and impose restrictive cost-sharing requirements [87]. Crucially, many Medicare Advantage enrollees report delaying dental care due to out-of-pocket costs [87], demonstrating how current designs perpetuate access disparities despite growing consensus that oral health is fundamental to healthy aging.

The U.S. is improving Medicare coverage through proposing dental care under Medicare [88] and covering dental work needed for medical treatments, such as preparing patients for organ transplants or cancer therapy [89]. These changes help connect dental and medical care—hospitals using both saw 22% better teamwork between providers. While not full dental coverage, they address the fact that 49% of Medicare users lacked dental care in 2023. Together, these policies test ways to expand access while building support for bigger reforms through practical examples of better health coordination.

Interprofessional education must also be strengthened, ensuring that medical, dental, and nursing students graduate with a shared understanding of geriatric oral health’s role in overall wellness [73,90]. Simulation-based training and joint clinical rotations can foster collaboration early in professional development [91]. Technological innovations hold immense potential to enhance monitoring and early intervention. Wearable sensors and smart toothbrushes could track oral hygiene habits in real time, alerting caregivers when assistance is needed [92]. AI-driven diagnostic tools, capable of analyzing intraoral images or risk factors, may empower primary care providers to identify oral cancers, infections, or periodontal disease during routine visits [93,94]. Furthermore, integrated electronic health records with interoperable dental and medical data would streamline communication across providers, reducing errors and improving care coordination [69].

Ultimately, progress will require commitment from policymakers, healthcare institutions, and educators of both integrated care delivery and coverage strategies to prioritize geriatric oral health as a public health imperative. By scaling proven models, investing in workforce training, and leveraging technology, the healthcare system can ensure that interdisciplinary oral care becomes the standard—not the exception—for aging populations worldwide. This shift will not only extend healthspan and reduce hospitalizations but also affirm that oral health is a fundamental component of dignity and well-being in later life [59].

## 7. Conclusions

Oral health is a critical component of healthy aging, yet it remains underprioritized in geriatric care. An interdisciplinary approach—uniting dentistry, medicine, nursing, nutrition, and caregiving—offers the most effective solution to address the complex needs of older adults. By breaking down silos between healthcare fields, improving professional education, and advocating for policy changes, we can enhance oral health outcomes and overall quality of life for aging populations. The future of geriatric oral care lies in collaboration, innovation, and a shared commitment to holistic well-being.

## Data Availability

Not applicable.
